# Metal-Ceramic Beads Based on Niobium and Alumina Produced by Alginate Gelation

**DOI:** 10.3390/ma14195483

**Published:** 2021-09-22

**Authors:** Enrico Storti, Marc Neumann, Tilo Zienert, Jana Hubálková, Christos Georgios Aneziris

**Affiliations:** Institute of Ceramics, Refractories and Composite Materials, TU Bergakademie Freiberg, Agricolastraße 17, 09599 Freiberg, Germany; Marc.Neumann@ikfvw.tu-freiberg.de (M.N.); Tilo.Zienert@ikfvw.tu-freiberg.de (T.Z.); Jana.Hubalkova@ikfvw.tu-freiberg.de (J.H.); aneziris@ikfvw.tu-freiberg.de (C.G.A.)

**Keywords:** metal-ceramic composites, alginate gelation, refractory metals, computed tomography, niobium

## Abstract

Full metal-ceramic composite beads containing different amounts of niobium and alumina, particularly 100 vol% alumina, 100 vol% niobium, and 95/5 vol% niobium/alumina, were produced by the alginate gelation process. The suspension for bead fabrication contained sodium alginate as gelling agent and was added dropwise into a calcium chloride solution to trigger the consolidation process. After debinding in air, sintering of the composite beads was performed under inert atmosphere. Samples in green and sintered state were analyzed by digital light microscopy and scanning electron microscopy equipped with energy dispersive X-ray spectroscopy. Investigations by mercury intrusion porosimetry revealed that pure alumina beads featured smaller pores compared to composite beads, although the open porosities were comparable. The fracture strength was evaluated on single beads. Contrary to the pure alumina, the composite beads showed a clear plastic deformation. Pure niobium beads showed a ductile behavior with very large deformations. XRD analyses revealed the presence of calcium hexaluminate and beta-alumina as minor phases in the alumina beads, while the composite ones contained about 25 wt% of impurities. The impurities comprised NbO arising from the oxidation, and β-Nb_2_C, from the reaction with the residual sodium alginate.

## 1. Introduction

Ceramic-metal composites benefit from the combination of high melting point, hardness and chemical stability of ceramics and high toughness and ductility of metals. Usually, the application temperature of such composites is limited by the relatively low melting point of the used metals or by the reaction between the metal and ceramic phase and/or environment. Regarding the melting point, so-called refractory metals, such as Zr, Mo, Nb, W, and Ta (to name the most abundant), can help extending the application to higher temperatures. In particular, niobium and tantalum exhibit a similar thermal expansion coefficient as alumina in a wide temperature range, which allows to produce composites with a high thermal shock resistance [1,2,3,4]. However, most studies on Nb-alumina composites are using very fine powders.

In a recent work, Zienert et al. reported for the first time the production of coarse-grained refractory composites consisting of refractory metals and refractory ceramic materials [5]. Refractory metals with a melting point above 2000 ${}^{{}^{\circ}}$C, namely Nb and Ta, were chosen and combined with alumina. Composites showed plastic deformation behavior between 1300 ${}^{{}^{\circ}}$C and 1500 ${}^{{}^{\circ}}$C [6]. The shrinkage of the coarse-grained refractory composites was clearly reduced in comparison to fine-grained materials, with the additional benefit of low residual stresses.

Especially in the case of ceramic materials, the ability to produce near-net shaped parts is very beneficial since it reduces the need for final machining, which is usually limited for sintered bodies (due to their brittleness) or often results in rejected green pieces (due to their weakness). Among these shaping methods, aqueous gel casting is one of the most common technique. In general, a ceramic suspension with high solid contents is mixed with an additive allowing for a direct sol-gel transition. Originally, toxic chemicals, such as acryl amide, were utilized as gelling agents [7,8,9,10]. Due to the toxicity of acryl amide, natural compounds are preferred today [11]. They are effective at low concentration, inexpensive, and environmentally friendly. Santacruz et al. obtained green densities higher than 60% of theoretical density and high green strength by gel casting of alumina suspensions, using concentrated agarose solutions [12]. It was demonstrated that gel casting with agar is also a good forming technique to produce net-shaped bodies in a very short time (consolidation takes <1 min) and with similar density values to those obtained by slip casting of nanosuspensions [13].

A particular class of natural compounds commonly used in gel casting is that of alginates, which are derived from brown seaweeds. They are widely applied as thickeners, stabilizers and gelling agents, especially in the food and pharmaceutical industry [14]. Alginates are water-dispersible salts of polysaccharides consisting of two essential monomers, 1,4-β-D-mannuronate (M) and 1,4-α-L-guluronate (G). Due to their biaxially linked structure, the combination of neighboring G monomers (so-called “G-blocks”) is of special interest for the gel-casting process. In particular, G-blocks enable the transition from an alginate sol to a stiff gel structure based on their buckled chain structure [15]. The process is based on the substitution of monovalent cations by divalent alkaline earth ions, such as Ca^2+^ or Ba^2+^, which results in the bonding between two G-blocks of different polysaccharide chain segments and leads to a 3D crosslinking and, thus, in the gelation. This phenomenon occurs stepwise and depends on the ratio between divalent ion (*M*^2+^) and G monomers [14]. The added alginates also provide a stabilizing effect. On the one hand, the viscosity increase and thickening effect result in a kinetic stabilization effect, hindering the sedimentation of dispersed particles in the suspension. On the other hand, alginates are natural polyelectrolytes that arrange around the solid particles leading to a steric stabilization effect. When free alkaline earth ions are supplied, the gelation takes place rather instantly, which can be exploited to manufacture granules or components with high specific surface area [16]. The shape and cross section of the manufactured product is determined by the nozzle through which the suspension is released and the gelation progress depends on the diffusion of alkaline earth ions into the structure core, limiting the achievable thickness. The suspension is usually pumped, poured, or dropped into a solution of alkaline earth salts, such as calcium chloride. This approach is especially useful in the food or pharmaceutical industry, for example, to encapsulate materials in alginate beads. However, technical applications, such as the gel casting of “spaghetti filters” for steel melt filtration, are possible, as well [17,18]. Alternatively, it can be beneficial to delay the gelation process by adding a chelating agent, which forms a complex with the calcium ions. By using an acid, calcium ions are then released from the complex and react with the alginate, finally forming a three-dimensional network. Silicon carbide bodies with high bending strength, good surface quality, and homogeneous microstructure were obtained with this approach [19].

Recently, hollow and full beads based on zirconia and steel were fabricated using the alginate gelation technique [20]. In particular, the authors combined austenitic stainless steel with transformation induced plasticity (TRIP) and/or twinning induced plasticity (TWIP) and partially stabilized zirconia reinforcing particles. The resulting samples exhibited high yield strength and high energy absorption capability, especially under compression, making them excellent candidates as crash absorber material. In addition, beads were spray coated in order to further improve the energy absorption in comparison to pure steel and uncoated MMC (metal-matrix composite) beads [21]. In the present work, a similar approach was used to produce full metal-ceramic beads based on alumina and niobium fine powders as starting materials. Such beads should serve as basis material for the manufacturing of coarse-grained refractory components with special functional properties, such as high electrical conductivity and improved thermal shock resistance, in comparison to traditional refractories.

## 2. Materials and Methods

### 2.1. Raw Materials

The main raw material for the beads production was niobium powder (Nb 99.95%, EWG Sondermetalle GmbH, Weissach-Flacht, Germany) with measured $d_{10}$, $d_{50}$, and $d_{90}$ of 9.2, 32.1, and 67.1 µm, respectively. Furthermore, fine calcined alumina (CT9FG, Almatis GmbH, Ludwigshafen, Germany) with measured $d_{10}$, $d_{50}$ and $d_{90}$ of 2.0, 5.5 and 20.6 µm, respectively, was also used. Sodium alginate powder (Carl Roth GmbH, Karlsruhe, Germany) worked as binder and enabled the gelation of the slurry. Liquid additives KM 1001 and KM 2000 (Zschimmer & Schwarz GmbH, Lahnstein, Germany) were required to reduce the water content of the slurry and provide sufficient stability. Finally, calcium chloride dihydrate CaCl_2_ · 2H_2_O (≥99%, Carl Roth GmbH, Germany) was used as solidifying agent.

### 2.2. Bead Production

The fabrication process was based on the gelation of alginate in contact with bivalent ions in an aqueous solution as solidifying agent. The raw powders (niobium powder, alumina and sodium alginate) were dry mixed for 5 min using a homogenizer DIAX 600 (Heidolph Instruments, Schwabach, Germany). Similarly, the liquid additives were dissolved in deionized water. Next, the two mixtures were filled into a polypropylene barrel together with alumina milling balls (3 cm in diameter) and mixed for 4 h. The formulations of the different slurries are given in Table 1. In the Nb_95 slurry, 5 vol% fine alumina was used in order to promote sintering of the samples, since the applied sintering temperature was relatively low compared to the melting point of Nb.

After mixing, the suspension was loaded into a 50 mL syringe and poured dropwise into a water solution (1 mass%) of calcium chloride from a height of approximately 3 cm. The bead formation occurred instantly. The process was automated by means of an infusion pump Perfusor Secura FT (Braun Melsungen AG, Melsungen, Germany), consisting of a computer-controlled motor turning a screw that pushes the plunger on the syringe. Different nozzle gauges allowed to control the size of the produced beads, as shown in Table 2. For simplicity, the three different sizes were labeled “L” (large), “M” (medium) and “S” (small), respectively. In general, thinner nozzles required a lower infusion rate in order not to exceed the maximum pressure applicable by the pump. It was not possible to use nozzles smaller than 21G (0.8 mm in diameter) without modifying the slurries, i.e., reducing the solids content.

**Figure 1:**
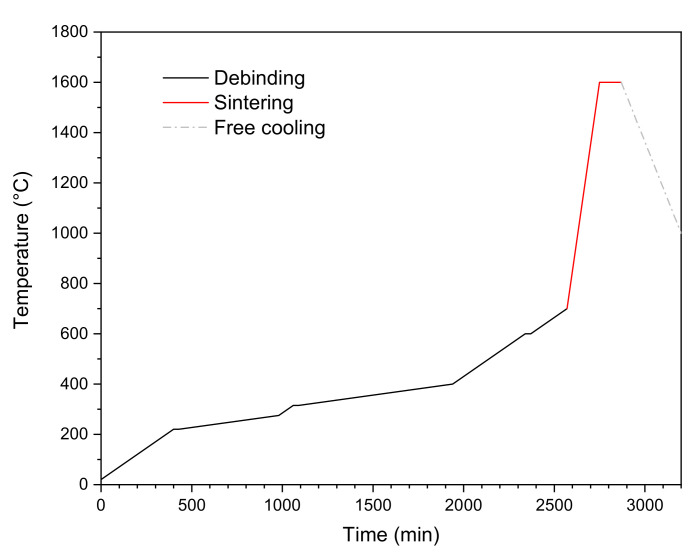
Combined debinding and sintering program for the pure alumina beads.

After gelation, the wet green beads were removed from the calcium chloride solution with the aid of a sieve, washed with deioinzed water to remove any solution residues, and, finally, dried at 50 ${}^{{}^{\circ}}$C for 24 h. It was reported that the decomposition of sodium alginate should be completed at approximately 680 ${}^{{}^{\circ}}$C, with three main exothermic peaks [20]. However, in the case of metal-ceramic composite beads, it was not possible to perform the debinding step up to this temperature, due to the high affinity of Nb to oxygen [5]. Instead, the following debinding program was chosen: 0.5 K/min heating rate up to 220 ${}^{{}^{\circ}}$C, 30 min dwell time at 220 ${}^{{}^{\circ}}$C, then 0.1 K/min up to 275 ${}^{{}^{\circ}}$C and 0.5 K/min to 300 ${}^{{}^{\circ}}$C. The furnace was then left to cool freely. With this schedule, only the first exothermic peak of sodium alginate was met. However, a preliminary debinding test showed some oxidation of Nb already at 300 ${}^{{}^{\circ}}$C. After debinding, the composite beads were sintered under inert atmosphere (Ar 5.0) inside an XGraphit furnace (XERION ADVANCED HEATING Ofentechnik GmbH, Berlin, Germany) with a heating rate of 5 K/min up to 1600 ${}^{{}^{\circ}}$C and a dwell time of 120 min at this temperature. Regarding the pure ceramic beads, a combined debinding and sintering program inside the same furnace was applied. In this case, three intermediate dwell steps were used, along with a final dwell time of 120 min at 1600 ${}^{{}^{\circ}}$C (as for the composite samples). The full schedule is shown in Figure 1.

### 2.3. Characterization

The rheological behavior of the slurries was evaluated by means of a rheometer HAAKE MARS 60 (Thermo Scientific, Waltham, MA, USA), using a coaxial cylinder measuring system with rotor CC38/Ti/SE and cup Z40. The measurements were performed under controlled-rate/steady-state conditions at a temperature of 20 ${}^{{}^{\circ}}$C. The shear rate was increased from 0 to 500 s^−1^, and it was then kept constant for 90 s and, finally, decreased again.

Mercury intrusion porosimetry was used to evaluate the open porosity and pore size distribution (PSD) of the sintered beads according to the International standard ISO/DIS 15901-1. The morphology and fracture surfaces of the samples were analyzed with aid of a digital light microscope (VHX-200 D, Keyence, Neu-Isenburg, Germany). The average diameter of each sample batch in dried, as well as in sintered, condition and the resulting shrinkage were estimated through image analysis on the micrographs. In addition, scanning electron microscopy coupled with EDS (ESEM XL30, FEI/Philips, Mainz, Germany) was used.

The fracture strength of the beads in sintered state was estimated with the aid of an universal testing machine TT2420 (TIRA GmbH, Germany) and a measuring test device for single beads equipped with a load cell of 1 kN. Thirty beads per batch were loaded to fracture in a diametral compression mode between two parallel steel plates at a displacement rate of 0.05 mm/s. The fracture stress was estimated from the force-displacement data according to the method reported by Kschinka et al., who investigated the mechanical behavior of glass spheres [22]:

(1) $$\sigma_{f}=\frac{2.8P}{\pi {d_{f}}^{2}},$$

where *P* is the load at fracture, and $d_{f}$ is the distance between the loading points at the instant of failure. $d_{f}$ was calculated for each single bead from the initial diameter, the crosshead speed, and the time to fracture. It should be pointed out here that the calculated strength is not equivalent to the stress obtained by a standard compression test on cylindrical samples. It was demonstrated that the tensile strength of brittle samples can be indeed determined by the compression test of irregular specimens [23]. After calculating the fracture strength, the failure probability was estimated for each sample, and the Weibull analysis was performed according to the European Standard DIN EN 843-5. Finally, the results were plotted in the double logarithmic coordinate system.

The 3D-macrostructure of selected large (L) beads was analyzed before and after mechanical loading using microfocus X-ray computed tomography (µ-CT). The analyses were performed with a CT-ALPHA (ProCon X-Ray GmbH, Sarstedt, Germany) equipped with a transmission X-ray tube FXE-160.20/25 (Feinfocus, Garbsen, Germany) and a flat panel X-ray detector Dexela 1512 (Perkin Elmer, Solingen, Germany). The µ-CT was operated with at 150 kV and 80 µA using a 0.3 mm copper filter in order to reduce beam hardening artifacts. The exposure time was set to 1 s for alumina samples and to 2 s for niobium containing samples. The volume data were reconstructed using the software Volex 6.0 (Fraunhofer EZRT, Fürth, Germany). The resulting voxel size was 5.5 µm for all investigated samples. The reconstructed volume data were visualized using the software VG Studio MAX 2.2 (Volume Graphics, Heidelberg, Germany).

Beads in sintered state were manually ground into fine powder and analyzed by means of XRD. The XRD diffractometer Empyrean (Malvern Panalytical GmbH, Kassel, Germany) was operated in Bragg-Brentano geometry, with standard Cu Kα radiation (λ = 1.54 Å) and a 2D detector. The measurements took place in the a 2θ-range from 10 to 140° with a scan step size of 0.0143° and a holding time of 160 s per step. The X-ray source was operated at 40 kV and 40 mA. The ICSD database was used to determine the phase composition of the samples in the HighScore Plus software version 4.8 (Malvern Panalytical GmbH, Germany). Rietveld refinement was applied for the quantitative analysis.

## 3. Results

**Figure 2:**
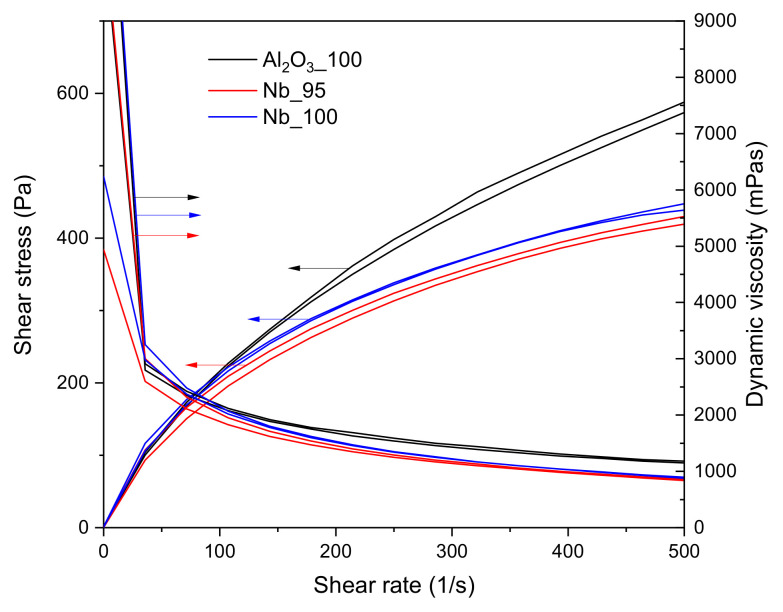
Rheological curves for all slurries.

The results of the rheological investigation are plotted in Figure 2. As expected, the slurries showed a clear shear thinning behavior up to 500 s^−1^, together with a slight thixotropy. The shear thinning behavior is a fundamental feature for slurries which need to be pumped or sprayed through a nozzle. Despite the lower solids content, the Al_2_O_3__100 slurry showed an overall higher dynamic viscosity compared to the others, hence requiring a higher shear stress to be stirred. This behavior was likely due to the particle size and morphology of the raw materials used in the preparation of the slurries: the tabular alumina had a $d_{90}$ of 20 µm, against the $d_{90}$ of 67 µm for the niobium powder. Due to the very similar composition, the Nb_95 and Nb_100 slurries showed almost identical rheological behavior.

**Figure 3:**
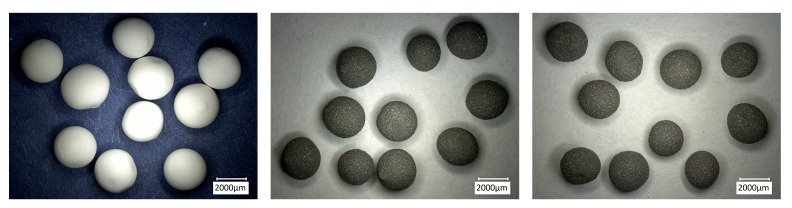
Beads in green state, after drying. Al_2_O_3__100-L (**left**), Nb_95-L (**center**), and Nb_100-L (**right**).

**Figure 4:**
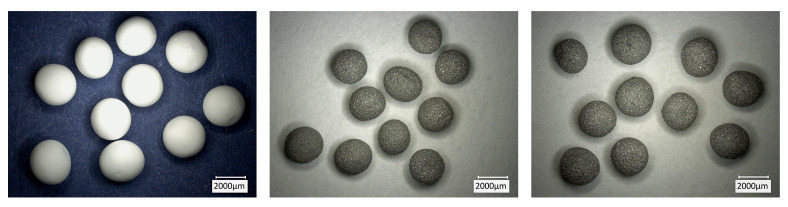
Beads in sintered state. Al_2_O_3__100-L (**left**), Nb_95-L (**center**), and Nb_100-L (**right**).

Figure 3 presents the large (L) beads in green state, after drying. It can be observed that samples from all batches had very similar size and shape. The use of nozzles allowed to decrease the average diameter and obtain almost perfect spheres. In addition, the metal-ceramic beads were comparatively smaller than the pure alumina ones, regardless of the nozzle used, as shown in Table 2. Since the syringes and nozzle-to-solution distance were not changed during the experiments, the size difference can be entirely attributed to the different rheological properties of the slurries. All beads showed a regular surface without defects, regardless of the composition. Large beads in sintered conditions are presented in Figure 4. Compared to the green state, a limited shrinkage was detected. Defects, such as pores or cracks, were not observed, indicating that the sintering and, especially, debinding schedules were effective. During sintering at 1600 ${}^{{}^{\circ}}$C, some niobium diffused from the samples into the alumina crucibles, which turned from white to gray. This is confirmed by the lighter gray shade of the composite beads after the thermal treatment. A strong diffusion of niobium along alumina grains was recently reported by Zienert et al. [5].

**Figure 5:**
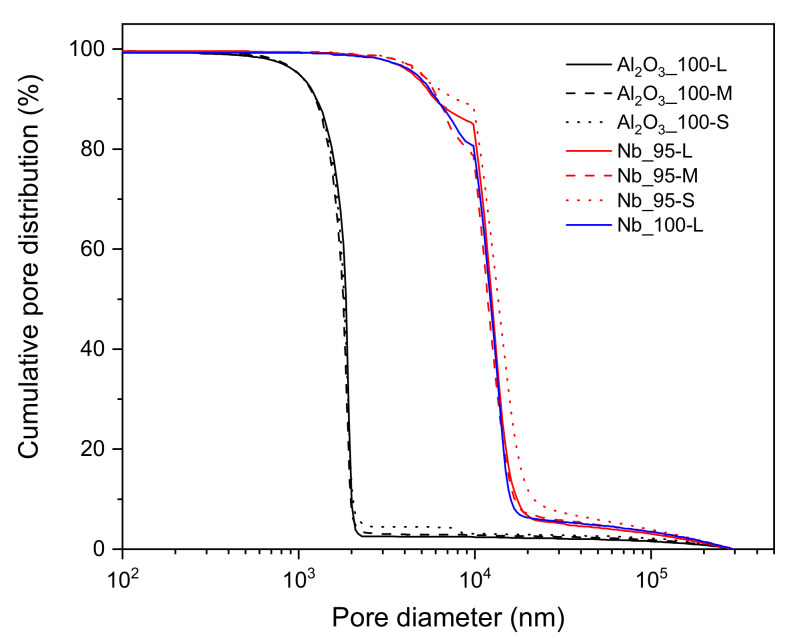
Normalized mercury intrusion curves for all batches.

The main results from mercury intrusion porosimetry are reported in Table 2. All batches showed an open porosity above 40% after sintering, which is quite high in comparison to common refractory ceramics. In addition, the larger beads generally had even higher porosity, as high as 47.1% for the Al_2_O_3__100-L batch. Larger beads have a smaller surface-to-volume ratio, which might result in less effective debinding and sintering steps. The apparent density obtained through mercury intrusion was similar for batches from the same slurry and with different sizes. As expected, the Nb_100-L batch showed a higher value of apparent density compared to the Nb_95-L one, due to the lack of alumina in the corresponding slurry. The mercury intrusion curves for all batches are presented in Figure 5. From these, several phenomena can be observed. First, alumina beads clearly featured smaller pores than Nb-based samples in general. The average pore size was about 1.8 µm for alumina batches and one order of magnitude higher (≈13 µm) for the Nb-containing ones. Since a diffusion rate close to zero can be assumed for niobium even at 1600 ${}^{{}^{\circ}}$C (which is only 0.68$\cdot T_{m}$ of Nb), it is obvious that sintering was much more effective for pure alumina beads. Second, the bead size had practically no impact on the pore size distribution of any batch. In particular, the smaller beads only showed slightly larger pores on average. Finally, the 5 vol% Al_2_O_3_ addition entailed no remarkable effect in the pore size distribution of large composite beads.

**Figure 6:**
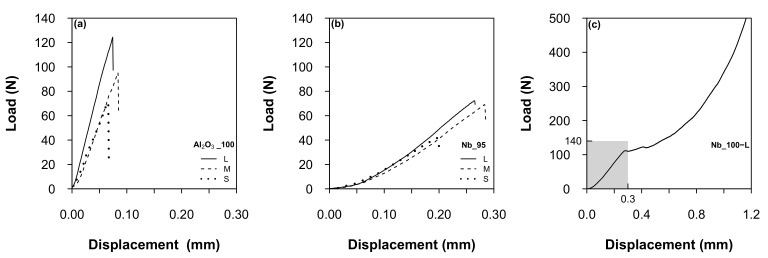
Selected load-displacement curves (compressive loading): (**a**) Al_2_O_3__100 batches; (**b**) Nb_95 batches; (**c**) Nb_100-L batch. The gray box represents the scale regions of (**a**), (**b**).

Some selected load-displacement curves for the different batches are presented in Figure 6. The fracture behavior of alumina beads was purely linear elastic, i.e., the curves were almost straight lines up to the point of fracture, at which the specimen broke into two hemispheres. In contrast, the composite beads did not separate at the point of fracture due to their high metallic content (see CT analysis below)). For comparison, only the linear elastic part of the force-displacement curves was taken into account for these batches. The confidence interval (not shown) was much narrower for the smaller alumina beads compared to the larger ones. In case of the composite beads, the confidence interval was quite narrow for any size. Observing Figure 6a,b, it is evident that the alumina beads broke at higher loads than the composite ones. On the other hand, the Nb-based samples reached much higher displacements, indicating a higher elastic compliance. Taking the size difference into account, all beads had similar strength levels (see Table 3). Finally, Figure 6c presents a selected load-displacement curve from the Nb_100-L batch. In this case, the samples showed a plastic behavior, with a clear plateau following the linear elastic region. Since it was not possible to identify a fracture point, no Weibull analysis was performed for the Nb_100-L batch.

**Figure 7:**
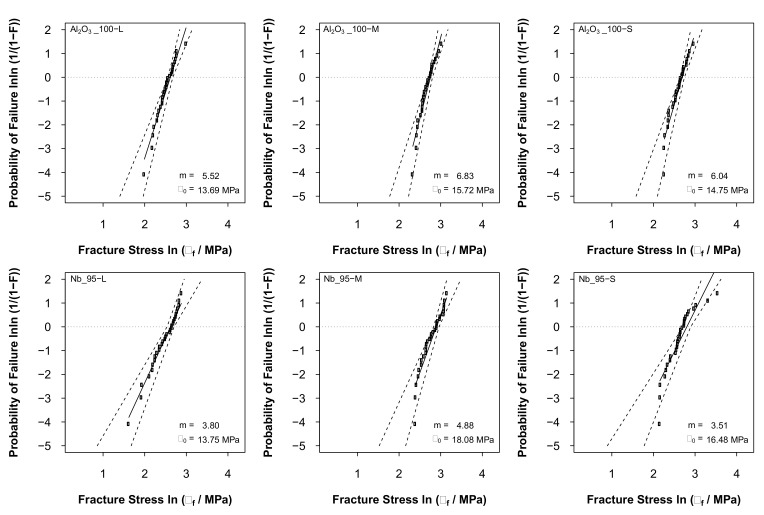
Weibull diagrams from the strength data (30 beads per batch). The dashed lines identify the 95% confidence interval.

The Weibull diagrams for all batches are presented in Figure 7. It was observed that the strength values were quite similar throughout all samples, regardless of the composition or size. As shown in Table 2, the bead diameters were all within the same order of magnitude; hence, no remarkable size effect was expected. In general, the Weibull parameter *m* was slightly higher for the alumina samples, as presented also in Table 3. The calculated values for *m* were within the expected range of partially sintered alumina [24]. The lower modulus *m* of the composite beads can be attributed to the interaction of two separate phases within the samples, which possess significantly different mechanical properties. However, the main factor was related to the average particle size and particle size distribution of the used raw materials. As mentioned in Section 2.1 and shown in the SEM images below, the alumina was much finer than niobium powder. In addition, the particle size distribution was not optimized to obtain the best possible packing density in the composite samples. As a result, the pure alumina beads showed better mechanical properties simply due to the finer particle size and better sintering activity of the material at 1600 ${}^{{}^{\circ}}$C.

The parameters of the fracture strengths obtained from the Weibull analysis are given in Table 3, according to the the European Standard DIN EN 843-5. In particular, $C_{l}$ and $C_{u}$ are the lower and upper limit of the 95% confidence interval for $\sigma_{0}$ (characteristic Weibull strength of the samples), respectively. Similarly, $D_{l}$ and $D_{u}$ are defined as the lower and upper limit of the 95% confidence interval for the Weibull modulus *m*.

**Figure 8:**
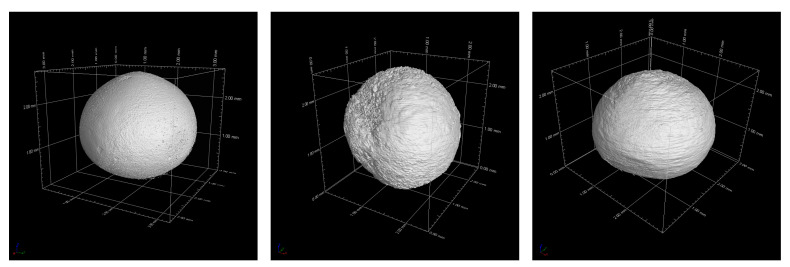
CT-rendered images of beads before compression test. Al_2_O_3__100-L (**left**), Nb_95-L (**center**), and Nb_100-L (**right**).

**Figure 9:**
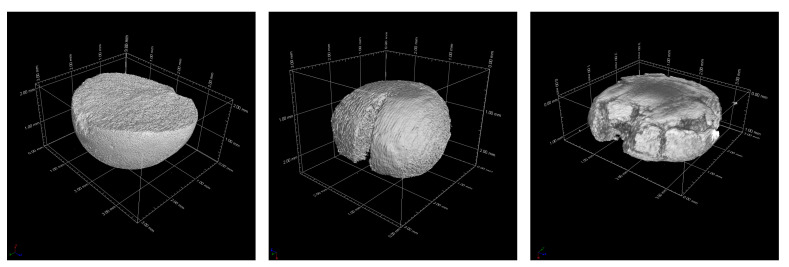
CT-rendered images of beads after compression test. Al_2_O_3__100-L (**left**), Nb_95-L (**center**), and Nb_100-L (**right**).

Reconstructed volume images from the CT analyses on the sintered beads are presented in Figure 8. The shape was quite close to that of a sphere, regardless of the composition. Pores can be observed on the Nb_95-L sample. On the other hand, in case of the pure alumina beads, the voxel size was larger than the average pore size (see Figure 5); hence, the samples appeared completely dense in this analysis. Three-dimensionally-rendered images of beads after the compression test are shown in Figure 9. As mentioned above, the alumina beads showed a clear linear elastic behavior: the Al${}_{2}$O${}_{3}$_100-L was clearly split into two hemispheres, typical for a brittle sample. From the analysis of multiple samples, it was confirmed that the beads were actually full. Hollow beads are also interesting and will be investigated in a future work, for instance, to decrease the total mass of the final components. The beads containing Nb behaved in a different way under load, and it was observed that they did not break catastrophically in two or more parts. Instead, after fracture, they kept deforming under compression until the test was manually terminated. The Nb_95-L bead showed a large deformation and vertical cracks, but its parts were still connected together. In case of the Nb_100-L sample, the deformation exceeded 50% without fragmentation. The use of such composite beads for the production of refractory components would likely result in a highly ductile character, as opposed to traditional ceramic parts.

**Figure 10:**
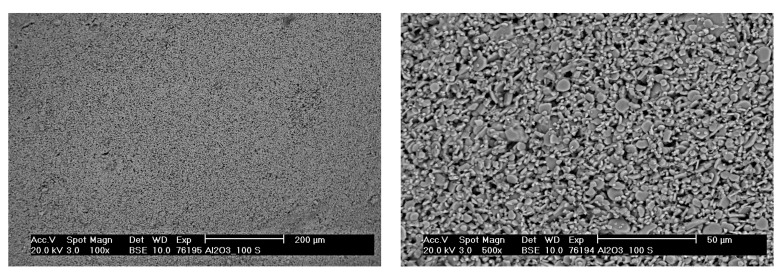
SEM (BSE mode) micrographs of Al${}_{2}$O${}_{3}$_100-S beads after sintering: overview (**left**) and detail (**right**).

**Figure 11:**
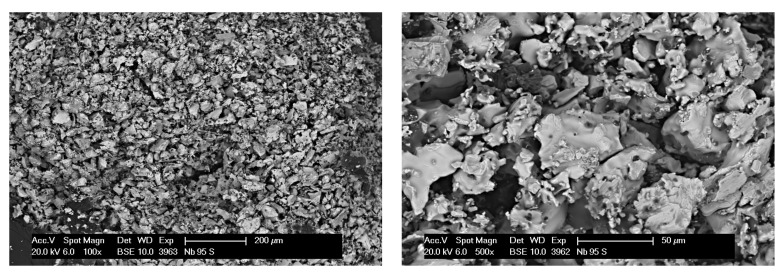
SEM (BSE mode) micrographs of Nb_95-S beads after sintering: overview (**left**) and detail (**right**).

SEM micrographs of the surface of pure alumina beads are presented in Figure 10. Samples of different sizes had very comparable microstructure; hence, only a Al${}_{2}$O${}_{3}$_100-S bead is shown here. A lot of pores were detected, as expected from the mercury intrusion porosimetry results. Overall, a regular microstructure with fine particles was observed. For the composite beads, a Nb_95-S bead is presented in Figure 11. The used magnifications are the same as in Figure 10, so the particle size difference is evident. Here, the use of a BSE detector allowed to clearly distinguish Nb and Nb-containing phases (such as carbides and oxides) from alumina, due to the remarkable difference in atomic number. In the micrograph, bright particles consisted mainly of Nb, while dark gray ones were identified as Al${}_{2}$O${}_{3}$ or similar. Overall, the alumina phase was well distributed within the Nb matrix as intended. In addition, it was observed that the Nb particles were only poorly sintered together. This was expected since the sintering temperature for the beads was only 0.68$\cdot T_{m}$ of Nb. The microstructure of Nb_100-L beads was similar, without the contrast from alumina grains. For this reason, micrographs of such samples are omitted here. EDS analysis on all composite beads revealed the presence of Nb, Al, O, and Ca. Calcium does not originate from the raw materials, but it was incorporated from the calcium chloride solution into the beads during the gelation phase.

**Figure 12:**
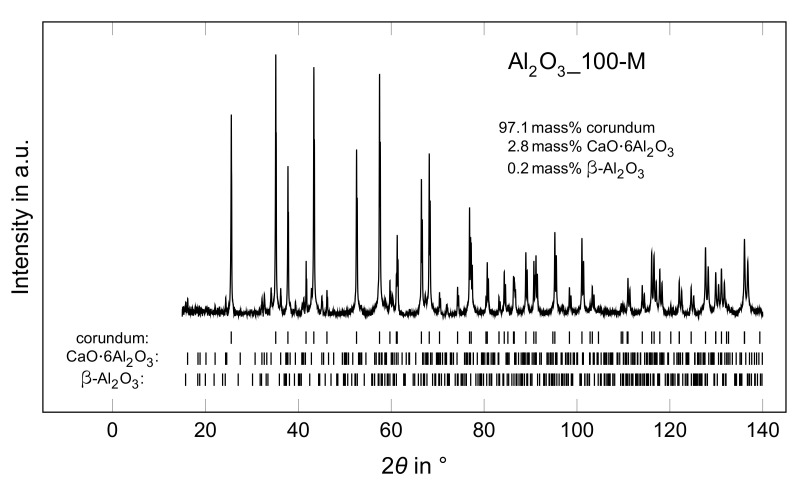
XRD pattern of Al${}_{2}$O${}_{3}$_100-M.

XRD analysis of the corundum powder revealed 98.9 mass% α-Al${}_{2}$O${}_{3}$ (ICSD 73725) with lattice parameters a=4.7594 Å and c=12.9921 Å, and 1.1 mass% of Na${}_{2}$O·11Al${}_{2}$O${}_{3}$ (depicted as β-alumina) [25] (ICSD 60635) with lattice parameters a=5.6026 Å and c=22.5166 Å. XRD results of Al${}_{2}$O${}_{3}$_100-M are shown in Figure 12. Only traces of β-alumina were found (≈0.2 mass%) in the Al${}_{2}$O${}_{3}$_100-M sample with lattice parameters a=5.5729 Å and c=22.4955 Å, whereas no β-alumina was observed for the Al${}_{2}$O${}_{3}$_100-L and -S beads. However, in all three samples, ≈3mass% CaO·6Al${}_{2}$O${}_{3}$ [26] (ICSD 202616, depicted as CA6) was formed with lattice parameters a=5.5587 Å and c=21.90 Å. Based on the smaller lattice parameters of β-alumina of the Al${}_{2}$O${}_{3}$_100-M sample in comparison to the corundum raw material, it can be assumed that this β-alumina underwent a chemical change during sintering and was incorporated into the lattice of CA6, which has a similar crystal structure.

**Figure 13:**
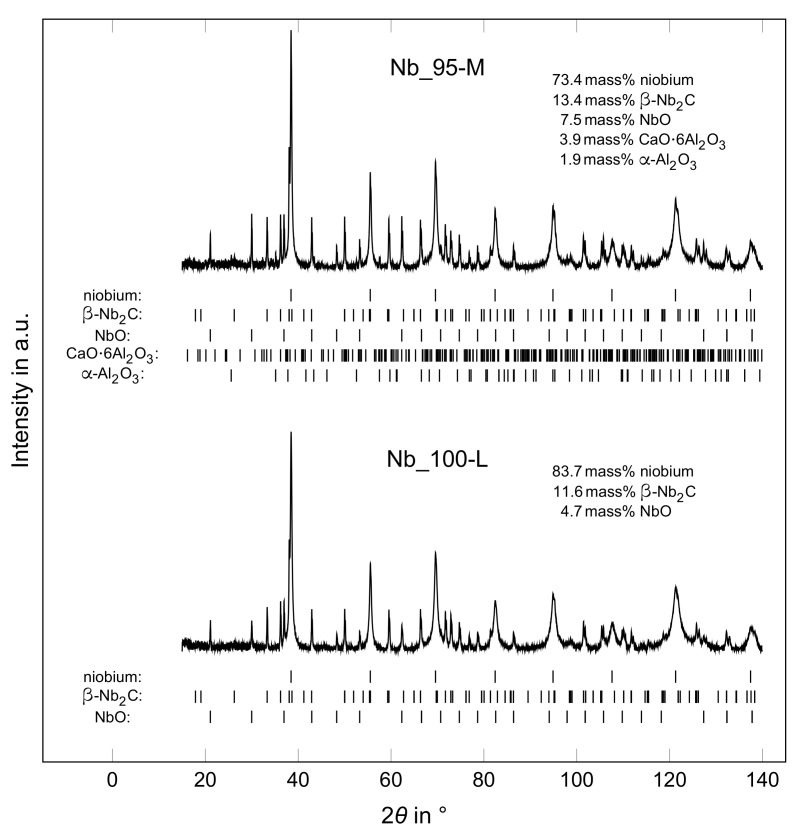
XRD patterns of Nb_95-M and Nb_100-L.

Besides niobium [27] (ICSD 76416) with lattice parameter a=3.3058 Å, ≈ 1.1 mass% β-Nb${}_{2}$C [28,29] (adopted from ICSD 33575) with lattice parameters a=5.38493 Å, and c=4.96897 Å, 2.3 mass% NbO [30] (ICSD 40318) with lattice parameter a=4.21246 Å and traces of at least one other phase were detected in the niobium raw material. The XRD pattern of the samples Nb_95-M and Nb_100-L are shown in Figure 13. After sintering the Nb_95 and Nb_100 samples, the lattice parameter of niobium slightly increased to a=3.3069 Å, which could be related to oxygen incorporation. In addition, the amount of β-Nb${}_{2}$C and NbO increased strongly to values between 13–21 mass% and 5–10 mass%, respectively. For the formation of niobium carbide, carbon was available from the alginate residues still present after the debinding step and also from the graphite crucibles in which the samples were enclosed during sintering. Lower amounts of secondary phases were found for the Nb_100-L sample. In all niobium-containing samples, the lattice parameters of β-Nb${}_{2}$C were lowered to a≈5.375 Å and c≈4.958 Å. The chemical composition of NbO was only slightly changed during annealing as the lattice parameter was with a=4.2104 Å always close to the original value. Only weak reflexes corresponding to 0.5–2 mass% α-Al${}_{2}$O${}_{3}$ and 0.5–4 mass% CaO·6Al${}_{2}$O${}_{3}$ were observed in the XRD patterns of the Nb_95 samples. Here, the lattice parameter *a* of CA6 remained almost the same as in the corundum raw material, whereas *c* increased to ≈21.92 Å.

## 4. Conclusions

In this study, metal-ceramic composite beads containing niobium and alumina, particularly 100 vol% alumina, 100 vol% niobium, and 95/5 vol% niobium/alumina, were produced by means of gel casting with sodium alginate as binder. The slurries were poured dropwise through nozzles with different diameters into a calcium chloride solution in order to obtain beads with distinct sizes. The samples containing Nb were carefully debound in air and subsequently sintered at 1600 ${}^{{}^{\circ}}$C under inert atmosphere to prevent the metal from oxidating.

Full beads with good spherical shape and average final diameters in the range 1.75–2.9 mm were obtained. The linear shrinkage after debinding and sintering was quite high, reaching more than 10% for the Al${}_{2}$O${}_{3}$_100-S batch. The open porosity obtained by mercury intrusion on the sintered beads amounted to more than 40% for all batches, with slightly higher values for the pure alumina samples. In addition, the intrusion curves revealed that pure alumina beads had a much smaller average pore size compared to the Nb-containing beads. This was likely related to the smaller grain size and, thus, higher sintering activity of alumina in contrast to the poor diffusivity of Nb at 1600 ${}^{{}^{\circ}}$C, since this sintering temperature was only equal to 0.68$\cdot T_{m}$. SEM investigations confirmed a more pronounced sintering of the pure alumina beads, with a more regular surface and finer pores. Under compression, the composite beads clearly showed a plastic behavior and achieved very high deformations without splitting, while the pure alumina ones failed catastrophically as expected. The compression tests allowed to estimate the fracture stress of the samples and to produce a Weibull plot. This was not possible in case of the pure Nb beads, due to the lack of a clear peak in the curves. The XRD analysis revealed the presence of calcium hexaluminate and of β-alumina in all batches, while NbO and β-Nb_2_C were found in the composite beads due to the reaction with the residual carbon and oxygen from the alginate binder.

This study demonstrated that the gel casting process allows to reliably produce spherical grains with defined properties, which can be used as aggregate fraction in new coarse-grained refractory castables, among others. Such products are being currently investigated and will be the object of an upcoming study. In addition, the particle size distribution of the slurries will be optimized to obtain the best possible particle packing, in order to improve the material densification during thermal treatment.

## Figures and Tables

**Table 1:** Composition of the slurries for beads production (values in mass%).

	Al_2_O_3__100	Nb_95	Nb_100
Materials			
CT9FG alumina	62.4	1.65	-
Nb powder	-	67.75	69.4
Sodium alginate	0.6	0.6	0.6
Deionized water	37	30	30
Additives (relative to solids)			
KM 1001	0.6	0.6	0.6
KM 2000	1	1	1

**Table 2:** Properties of the beads before and after thermal treatment. “L” = Large; “M” = Medium; “S” = Small.

		Batch						
**Property**	**Unit**	**Al_2_O_3__100-L**	**Al_2_O_3__100-M**	**Al_2_O_3__100-S**	**Nb_95-L**	**Nb_95-M**	**Nb_95-S**	**Nb_100-L**
Nozzle diameter	mm	no nozzle	1.2	0.8	no nozzle	1.2	0.8	no nozzle
Bead diameter (dried)	mm	3.01 ± 0.10	2.59 ± 0.11	2.42 ± 0.09	2.71 ± 0.15	2.12 ± 0.05	1.79 ± 0.06	2.69 ± 0.16
Bead diameter (sintered)	mm	2.91 ± 0.09	2.42 ± 0.09	2.15 ± 0.06	2.47 ± 0.11	1.97 ± 0.10	1.75 ± 0.06	2.60 ± 0.13
Shrinkage	%	3.3	6.7	11.4	8.6	6.9	2.1	3.5
Open porosity	%	47.1	46.0	45.5	43.1	42.2	42.5	42.5
Apparent density	g/cm^3^	3.98	3.95	3.93	6.58	6.85	6.54	7.24

**Table 3:** Characteristics of the Weibull distribution, as defined in the European Standard DIN EN 843-5.

	*m*	$D_{u}$	$D_{l}$	$\sigma_{0}$ (MPa)	$C_{u}$ (MPa)	$C_{l}$ (MPa)
Al_2_O_3__100-L	5.52	7.05	4.33	13.69	14.52	12.92
Al_2_O_3__100-M	6.83	8.73	5.36	15.72	16.48	15.00
Al_2_O_3__100-S	6.04	7.72	4.74	14.75	15.56	13.99
Nb_95-L	3.80	4.86	2.98	13.75	14.97	12.64
Nb_95-M	4.88	6.24	3.83	18.08	19.31	16.93
Nb_95-S	3.51	4.48	2.75	16.48	18.07	15.05

## Data Availability

Not applicable.
